# Structure-Based Peptide Inhibitor Design of Amyloid-β Aggregation

**DOI:** 10.3389/fnmol.2019.00054

**Published:** 2019-03-04

**Authors:** Jinxia Lu, Qin Cao, Chuchu Wang, Jing Zheng, Feng Luo, Jingfei Xie, Yichen Li, Xiaojuan Ma, Lin He, David Eisenberg, James Nowick, Lin Jiang, Dan Li

**Affiliations:** ^1^Key Laboratory for the Genetics of Developmental and Neuropsychiatric Disorders (Ministry of Education), Bio-X Institutes, Shanghai Jiao Tong University, Shanghai, China; ^2^UCLA-DOE Institute for Genomics and Proteomics, University of California, Los Angeles, Los Angeles, CA, United States; ^3^Interdisciplinary Research Center on Biology and Chemistry, Shanghai Institute of Organic Chemistry, Chinese Academy of Sciences, Shanghai, China; ^4^University of Chinese Academy of Sciences, Beijing, China; ^5^Shanghai Center for Women and Children’s Health, Shanghai, China; ^6^Department of Chemistry, University of California, Irvine, Irvine, CA, United States; ^7^Department of Neurology, Easton Center for Alzheimer’s Disease Research, David Geffen School of Medicine, University of California, Los Angeles, Los Angeles, CA, United States

**Keywords:** neurodegenerative diseases, Alzheimer’s disease, Aβ fibril, protein misfolding, structure-based inhibitor design

## Abstract

Many human neurodegenerative diseases are associated with amyloid fibril formation. Inhibition of amyloid formation is of importance for therapeutics of the related diseases. However, the development of selective potent amyloid inhibitors remains challenging. Here based on the structures of amyloid β (Aβ) fibrils and their amyloid-forming segments, we designed a series of peptide inhibitors using RosettaDesign. We further utilized a chemical scaffold to constrain the designed peptides into β-strand conformation, which significantly improves the potency of the inhibitors against Aβ aggregation and toxicity. Furthermore, we show that by targeting different Aβ segments, the designed peptide inhibitors can selectively recognize different species of Aβ. Our study developed an approach that combines the structure-based rational design with chemical modification for the development of amyloid inhibitors, which could be applied to the development of therapeutics for different amyloid-related diseases.

## Introduction

Amyloid diseases, including many neurodegenerative diseases, are increasingly prevalent in aging societies (Eisenberg and Jucker, 2012; Dobson, 2017). The pathogenesis of these devastating diseases is closely associated with aberrant protein aggregation (Chiti and Dobson, 2006). In the progression of amyloid aggregation, soluble proteins undergo a series of conformational changes and self-assemble into insoluble amyloid fibrils (Riek and Eisenberg, 2016). Plaques containing amyloid fibrils are one of the histological hallmarks of Alzheimer’s and Parkinson’s diseases (Lee et al., 1991; Spillantini et al., 1997; Koo et al., 1999). Various strategies have been exploited to interfere with the process of amyloid aggregation by targeting different conformational species, including stabilizing monomers by antibodies (Ladiwala et al., 2012), redirecting monomers to nontoxic off-pathway oligomers by polyphenolic compounds (Ehrnhoefer et al., 2008), accelerating mature fibril formation by fibril binders (Bieschke et al., 2012; Jiang et al., 2013), inhibiting fibril growing by peptide blockers (Seidler et al., 2018), and disrupting amyloid assembly by nanomaterials (Hamley, 2012; Huang et al., 2014; Lee et al., 2014; Li et al., 2018; Han and He, 2018). Many of these strategies show promising inhibitory effects against toxic amyloid aggregation (Härd and Lendel, 2012; Arosio et al., 2014), but so far none has led to clinical drugs because of unsettled issues such as target selectivity, side effects, membrane permeability and penetration of the blood-brain barrier.

Amyloid β (Aβ) has long been targeted for drug development and therapeutic treatment of Alzheimer’s disease (Caputo and Salama, 1989; Haass and Selkoe, 2007; Sevigny et al., 2016). In addition to the common difficulties in targeting amyloid proteins, Aβ is especially challenging since it contains multiple species with various lengths generated by γ-secretases (Acx et al., 2014; Kummer and Heneka, 2014; Szaruga et al., 2017). Many studies have shown that Aβ_42_ rather than Aβ_40_ is more prone to form toxic aggregates, and the ratio of Aβ_42_/Aβ_40_ is better correlated with the pathology rather than the amount of each individual Aβ species (Lewczuk et al., 2004; Jan et al., 2008; Kuperstein et al., 2010). However, selective inhibition of Aβ_42_ is very difficult because it is only two residues longer than Aβ_40_ at the C-terminus. In this work, we targeted two key amyloid-forming segments of Aβ_42_ (^16^KLVFFA^21^ and ^37^GGVVIA^42^) based on the cryo-EM structure of Aβ_42_ fibrils reported recently (Gremer et al., 2017). We designed peptide binders of these two segments using RosettaDesign with the atomic structures of these two segments as templates (Sawaya et al., 2007; Colletier et al., 2011). The designed sequences showed inhibitory effect to Aβ_42_ fibril formation. We further utilized a macrocyclic β-sheet mimic scaffold (Zheng et al., 2011; Cheng et al., 2012, 2013) to constrain the designed peptide inhibitors in β-conformation, which significantly enhanced the inhibitory effect on Aβ_42_ aggregation. Furthermore, we show that the peptide inhibitor designed to target the C-terminus of Aβ_42_ can selectively inhibit Aβ_42_ aggregation, but not to that of Aβ_40_ or other amyloid proteins. Our work shed light on the application of structure-based rational design combined with chemical modification in the development of therapeutics for Alzheimer’s disease and other amyloid-related diseases.

## Materials and Methods

### Structure-Based Design by Rosetta Software Package

#### Initial Structure Model for Design

We chose two key amyloidogenic Aβ segments, ^16^KLVFFA^21^ and ^37^GGVVIA^42^, for our inhibitor design. The design templates were taken from the crystal structures of KLVFFA (PDB ID: 2Y2A) and GGVVIA (PDB ID: 2ONV). The backbone of the inhibiting pentapeptide was fully extended to mimic β-conformation. This extended peptide was aligned with the N, C, and O backbone atoms of the template.

#### Rosetta Design of Fibril-Inhibiting Peptides

The peptide inhibitors were subsequently designed to ensure maximal interaction, while keeping the template amino acid sequence fixed. Computational designs were carried out using the RosettaDesign software package^1^. This algorithm involves building side-chain rotamers of all L-amino acids onto a fixed peptide backbone. The optimal set of side-chain rotamers at each position with the best interaction energy is then identified, with the guidance of a full-atom energy function containing a Lennard-Jones potential, an orientation-dependent hydrogen bond potential, an implicit solvation term, amino acid-dependent reference energies, and a statistical torsional potential that depends on the backbone and side-chain dihedral angles. Finally, the entire structure was refined by simultaneously optimizing degrees of freedom on: (1) the rigid-body geometry between the inhibiting peptide and template; (2) backbone torsions of each peptide; and (3) side chain torsions of each peptide. The lowest-energy model was picked and the interaction energies of each final model from different peptide inhibitors are listed in Table 1.

**Table 1:** Characteristics of designed peptide inhibitors.

Targeting sequence	Aβ target	Inhibitor ID	Inhibitor sequence	Predicted binding energy (kcal/mol)	Buried area (Å^2^)	Shape complementarity (Sc; Lawrence and Colman, 1993)
^16^KLVFFA^21^	Aβ_42_	K6A1	TLWYK	−16	280	0.65
	Aβ_40_	K6A2	EHWYH	−13	278	0.7
		G6A1	HYFKY	−19	271	0.67
^37^GGVVIA^42^	Aβ_42_	G6A2	HYYIK	−15	252	0.72
		G6A3	KYYEI	−14	270	0.66

### Circular Dichroism Spectroscopy (CD)

Chirascan spectrometer (Applied Photophysics) equipped with a Peltier temperature controller (Quantum Northwest) is used to acquire the CD spectra. Far UV spectra (240–180 nm) are collected in 0.05 cm path-length quartz cells. Sample concentration is 600 μM. All measurements are conducted at 23°C. Water is used as blank for subtraction from corresponding samples. Secondary structure is predicted from CD using CDPro (Eisenberg and Jucker, 2012).

### Preparation of Aβ_42_ and Aβ_40_

Both Aβ_42_ and Aβ_40_ were purified from *E. coli* expression system as reported previously (Dobson, 2017). The expression constructs contain an N-terminal His-tag, followed by 19 repeats of Asn-Ala-Asn-Pro, the Tobacco etch virus (TEV) protease site, and the sequence of Aβ_42_ or Aβ_40_. Purification of Aβ_42_ and Aβ_40_ follows the same experimental procedure. Briefly, the Aβ fusion protein was overexpressed into inclusion bodies in *E. coli* BL21(DE3) cells. The inclusion bodies were solubilized in 8 M urea, followed by washing in a high salt and detergent-containing solution. The Aβ fusion proteins were purified through HisTrap^TM^ HP Columns, followed by reversed-phase high-performance liquid chromatography (RP-HPLC). After cleavage by TEV protease, Aβ was released from fusion protein, and purified through RP-HPLC followed by lyophilization. To disrupt preformed Aβ aggregates, lyophilized Aβ powder was resuspended in 100% HFIP and incubated at room temperature for 2 h. HFIP was fully removed by evaporation. Before used in ThT or MTT assay, Aβ was freshly dissolved in 10 mM NaOH, solubilized by sonication. Aβ is further diluted to 200 μM in phosphate buffer saline (PBS) as a stock solution.

### Synthesis of Designed Macrocyclic Peptides

Designed macrocyclic peptides were synthesized by standard Fmoc solid-phase peptide synthesis. In brief, with Boc-Orn(Fmoc)-OH attached onto 2-chlorotrityl chloride resin, the linear peptide was elongated by standard automated Fmoc solid-phase peptide synthesis. Then, the peptide was cleaved from the resin under mildly acidic conditions, followed by being cyclized to the corresponding protected cyclic peptide by slow addition to HCTU and DIEA in dilute (ca. 0.5 mM) DMF solution. Since the C-terminus of the protected linear peptide comprises an amino acid carbamate (Boc-NH-CHR-COOH), the cyclization condition efficiently avoids problematic epimerization. The final deprotection with TFA solution followed by RP-HPLC purification yielded macrocyclic peptides in 18%–43% overall yield, based on the loading of Boc- Orn(Fmoc)-OH attached onto the resin.

### ^1^H NMR Spectroscopy

^1^H NMR experiments for the designed macrocyclic peptides were performed in D_2_O with the internal standard 4,4-Dimethyl-4-silapentane-1-ammonium trifluoroacetate (DSA) at 500 MHz (Brüker Avance) or 600 MHz (Brüker Avance). All peptides were studied at 2 mM in D_2_O at 298 K. Sample solutions were prepared gravimetrically by dissolving the macrocyclic peptides directly in solvent. All amino groups were assumed to be protonated as the TFA salts for molecular weight calculation. The data were processed with the Brüker XwinNMR software.

### ThT Fluorescence Assay

Thioflavin T (ThT) fluorescence assays were performed to monitor the real-time aggregation of Aβ_42_ and Aβ_40_ in the absence or presence of designed peptides. ThT assays were conducted in 96-well plates (black with flat optical bottom) in a Varioskan fluorescence plate reader (Thermo Scientific, 444 nm excitation, 484 nm emission). Each experiment was run in triplicates. The reaction solution contained 30 μM pre-disaggregated Aβ_42_ or Aβ_40_, 10 μM ThT, and designed peptides at indicated concentrations in PBS. The ThT assay was conducted at 37°C, without shaking for the Aβ_42_ aggregation assay, and with shaking (300 rpm) for Aβ_40_ aggregation assay. The fluorescence readings were collected every 2 min.

### Native Gel Electrophoresis

Purified Aβ_42_ powder was pre-treated by HFIP and dissolved in PBS buffer as described above. Aβ_42_ solution was diluted to a final concentration of 10 μM with or without the macrocyclic peptides mcG6A1, mcG6A2, and mcK6A1 (the final concentration of the inhibitors was 50 μM), and incubated at 37°C for 7.5 h. The samples were separated by a NativePAGE 4%–16% BisTris Gel (Novex, USA) and transferred to a nitrocellulose membrane pre-packed in iBlot 2 NC Mini Stacks (Novex, USA) by iBlot 2 Dry Blotting System (Life technologies, USA). The membrane was probed by β amyloid, 1–16 (6E10) Monoclonal Antibody (Covance, USA) and secondary anti-mouse IgG-HRP (MBL, USA), and detected with SuperSignal West Pico Chemiluminescent Substrate (Thermo, USA). The freshly made Aβ_42_ sample without inhibitors was loaded to a separated native gel and detected by the same method as a 0-h control. The molecular weight of the protein aggregates or monomer were accurately determined by the protein standard especially for native gel (Life technologies; cat. # LC0725).

### Transmission Electron Microscopy (TEM)

For specimen preparation, 5 μl of each sample was deposited onto a glow-discharged carbon film on 400 mesh copper grids, followed by washing in water twice. The grids were then stained in 0.75% uranyl formate. A Tecnai G2 Spirit transmission electron microscope operating at an accelerating voltage of 120 kV was used to examine and visualize the samples. Images were collected by a 4k × 4k charge-coupled device camera (BM-Eagle, FEI).

### Cell Viability Assay

We performed MTT-based cell viability assays to evaluate the toxicity of Aβ_42_ in the absence or presence of the designed peptides. We used a CellTiter 96 aqueous non-radioactive cell proliferation assay kit (Promega cat. # G4100). PC-12 cell lines (ATCC; cat. # CRL-1721) were used to test the cytotoxicity of Aβ_42_ under different conditions. PC-12 cells were cultured in ATCC-formulated RPMI 1640 medium (ATCC; cat. # 30-2001) with 5% fetal bovine serum and 10% heat-inactivated horse serum. Before the cell viability experiment, PC-12 cells were plated at 10,000 cells per well in 96-well plates (Costar; cat. # 3596), and cultured for 20 h at 37°C in 5% CO_2_. For the preparation of Aβ_42_ and peptide inhibitors mixture solutions, purified and pre-disaggregated Aβ_42_ samples were dissolved in PBS to a final concentration of 5 μM, followed by the addition of different peptide inhibitors at indicated concentrations. The mixture solution was filtered through a 0.22 μm filter, followed by incubation at 37°C without shaking for 16 h. To initiate the cell viability assay, 10 μl of pre-incubated mixture was added to each well containing 90 μl medium. After incubation at 37°C in 5% CO_2_ for 24 h. Fifteen microliter Dye solution (Promega; cat. # G4102) was applied into each well. After incubation for 4 h at 37°C, 100 μl solubilization Solution/Stop Mix (Promega; cat. # G4101) were added. After further incubation at room temperature for 12 h, the absorbance reading was collected at 570 nm with background reading at 700 nm. Four replicates were measured in parallel for each sample. The cell survival rate was normalized by using the PBS-treated cells as 100% and 0.02% SDS-treated cells as 0% viability.

## Results

### Structure-Based Design of Peptide Inhibitors

**Figure 1:**
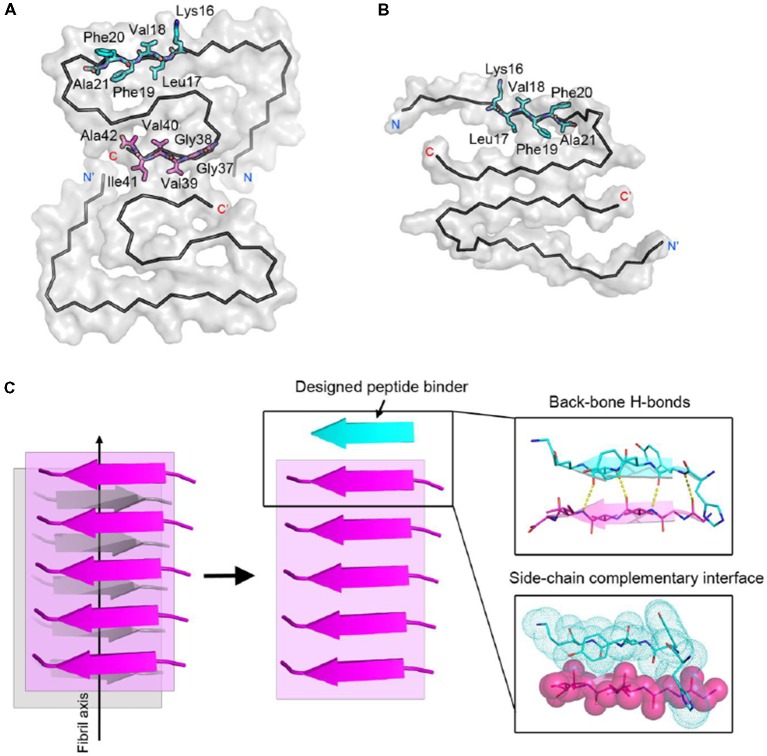
Structure-based peptide inhibitor design of amyloid β (Aβ) amyloid aggregation. **(A)** The fibril structure of full-length Aβ_42_ determined by cryo-EM (PDB ID: 5OQV) is shown as black ribbons. The atomic crystal structures of peptides KLVFFA (PDB ID: 2Y2A, cyan) and GGVVIA (PDB ID: 2ONV, magenta) are aligned on one of the two protofilaments of the full-length Aβ_42_ fibril structure and shown as sticks. **(B)** The fibril structure of full-length Aβ_40_ determined by solid-state NMR (PDB ID: 2LMN) is shown as black ribbons. The atomic crystal structure of peptides KLVFFA is aligned on one of the two protofilaments of the full-length Aβ_40_ fibril structure and shown as sticks.** (C)** Design strategy for peptide inhibitors of amyloid fibrils. The designing template is a five-stranded sheet extracted from the fibrillar structure of the targeting segment. Peptide inhibitors (in cyan) are designed to have the optimal interactions with the target *via* backbone hydrogen bonds (yellow dashed lines) and complementary side-chain interactions (shown as spheres and dots). Oxygen atoms are in red. Nitrogen atoms are in blue.

To effectively inhibit Aβ fibril formation, we targeted two key amyloid-forming segments of Aβ_42_: ^16^KLVFFA^21^ and ^37^GGVVIA^42^ (Figure 1A). The ^16^KLVFFA^21^ segment has been identified as a key segment accounting for both Aβ_42_ and Aβ_40_ nucleation and fibrillation (Ahmed et al., 2010; Colletier et al., 2011; Fawzi et al., 2011; Lu et al., 2013). In the known structures of Aβ fibrils including the recent cryo-EM structure of Aβ_42_ and the previous solid-state NMR structure of Aβ_40_ (Paravastu et al., 2008; Ahmed et al., 2010), this segment forms extended β-strands and stacks repetitively along the fibril axis to form the Aβ fibril core (Supplementary Figure S1). Thus, we selected ^16^KLVFFA^21^ as one of our design targets. In addition, the cryo-EM structure of Aβ_42_ fibril shows that the C-terminal segment ^37^GGVVIA^42^ plays an essential role in the fibril formation (Supplementary Figure S2). ^37^GGVVIA^42^ of one protofilament interdigitates *via* side chains with its counterpart of the neighboring protofilament forming a steric-zipper-like interaction to compose the mature fibril. Therefore, preventing the self-assembly of either ^16^KLVFFA^21^ or ^37^GGVVIA^42^ may potentially inhibit the assembly of Aβ_42_ fibrils.

For structure-based computational design, we used the atomic structures of ^16^KLVFFA^21^ (PDB ID: 2Y2A) and ^37^GGVVIA^42^ (PDB ID: 2ONV) as templates. The atomic structures of these two segments represent their conformations in the context of the full-length Aβ fibrils (Figures 1A,B). Based on the structures of the two targeting templates, we designed pentapeptides that bind the targeting segments to block the stacking of Aβ molecules along the fibril axis, thus inhibiting fibril growth (Figure 1C). We extracted a five-stranded layer from the steric-zipper structure of each segment, and docked a fully extended pentapeptide backbone on one end of the β-sheet. Then, we maximized the backbone interaction with the template by forming a backbone H-bonding network. To further increase the binding affinity and selectivity, we searched for the canonical L-amino acids at each position of the pentapeptide, using RosettaDesign (Leaver-Fay et al., 2011) for the side chains and their conformations, that provide maximal interactions with the template.

**Figure 2:**
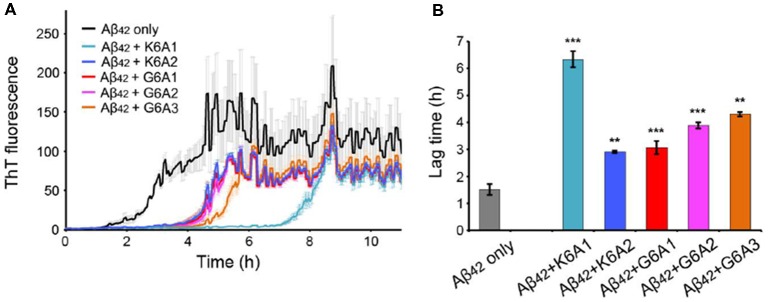
Inhibitory effects of designed peptides on Aβ_42_ amyloid aggregation measured by the thioflavin T (ThT) fluorescence assay. **(A)** The ThT fluorescence curves of Aβ_42_ in the presence of designed peptide inhibitors. The molar ratio of Aβ:peptide-inhibitor is 1:5. Three replicates were measured for each curve. The lag time of Aβ_42_ aggregation in the presence of peptide inhibitors is compared in **(B)**. *p-value < 0.05; **p-value < 0.01; ***p-value < 0.001.

Next, we calculated the binding energy, buried surface area and shape complementarity of the binding interfaces of the predicted binding models, and proceeded with experimental validation for the top-ranking designs. Using ThT fluorescence assay, we observed that the top-5 designs showed inhibitory effects on Aβ_42_ amyloid aggregation by significantly delaying the aggregation lag time (Xue et al., 2008; Knowles et al., 2009; Figure 2). Among them, two peptide inhibitors (K6A1 and K6A2) were designed for targeting ^16^KLVFFA^21^ and three (G6A1-G6A3) were for ^37^GGVVIA^42^ (Table 1). Furthermore, unlike their targeting segments, the five designed peptides do not form amyloid fibrils by themselves (Supplementary Figure S3).

### Constraining the Structures of Designed Peptides With a Chemical Scaffold

**Figure 3:**
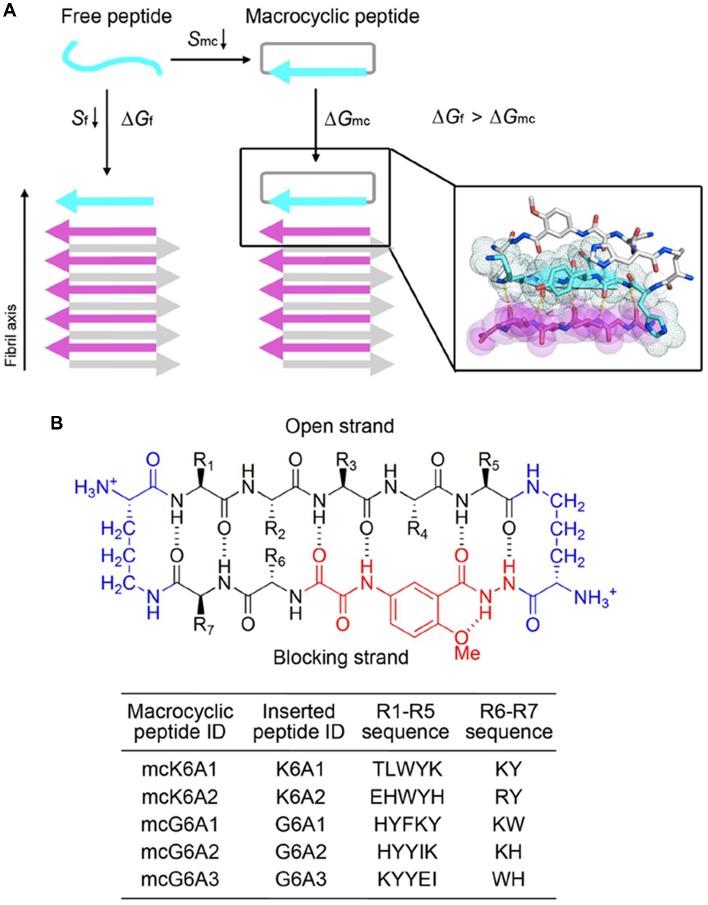
Design of macrocyclic peptide inhibitors. **(A)** The schematic shows that as the macrocyclic β-sheet mimic scaffold constrains the designed peptide sequence into a β-strand, the entropy loss is diminished during the process of target binding. “f” represents free peptide; “mc” represents macrocyclic peptide. The zoom-in view shows the structure model of a macrocyclic inhibitor binding to the targeting segment. The targeting segment is in magenta. The designed sequence is in cyan. The macrocyclic scaffold is in gray. H-bonds between the designed sequence and the targeting sequence are labeled by yellow dotted lines. **(B)** The 42-membered macrocyclic scaffold used in this study. The open strand (positions R1 to R5) accommodates the designed peptides in β-conformation. Two δ-linked ornithine turn units are in blue. The Hao unit in the blocking strand is in red. Sequences of R1-R7 are listed in the table below.

We next sought to enhance the potency of the peptide inhibitors. In our design, the peptide inhibitors were expected to adopt an extended β-strand conformation to maximize the interaction with the template (Figure 1C). However, in solution, the peptides are mainly unstructured (Supplementary Table S1). Thus, upon binding to the template, the peptides need to undergo conformational change to form extended β strands, which causes an entropy decrease and thus weakens the binding affinity of the peptides to the template. To overcome the entropy lost during the conformational change, we adopted a macrocyclic β-sheet mimic scaffold to fix the peptide binders into β strands (Figure 3A). The Nowick group has developed a series of macrocycles in different sizes as robust scaffolds for displaying peptides of interest in β-conformation (Liu et al., 2012; Cheng et al., 2013; Salveson et al., 2016; Kreutzer et al., 2017). According to the length of our designed peptides, we chose a 42-membered macrocyclic β-sheet mimic and grafted the designed sequence into the open strand of the macrocyclic scaffold with appropriate amino acids in the blocking strand for proper solubility and stability (Figure 3B). The β-strand conformation of the grafted sequence was validated by measuring the α-H shifts and δOrn anisotropy using ^1^H NMR experiments (Supplementary Figures S4, S5) in solution. Furthermore, we confirmed that the macrocycles carrying the designed peptides do not form amyloid aggregation in solution, while those carrying native amyloid-forming sequences may form amyloid fibrils with an out-of-register packing (Lu et al., 2013; Supplementary Figure S3).

**Figure 4:**
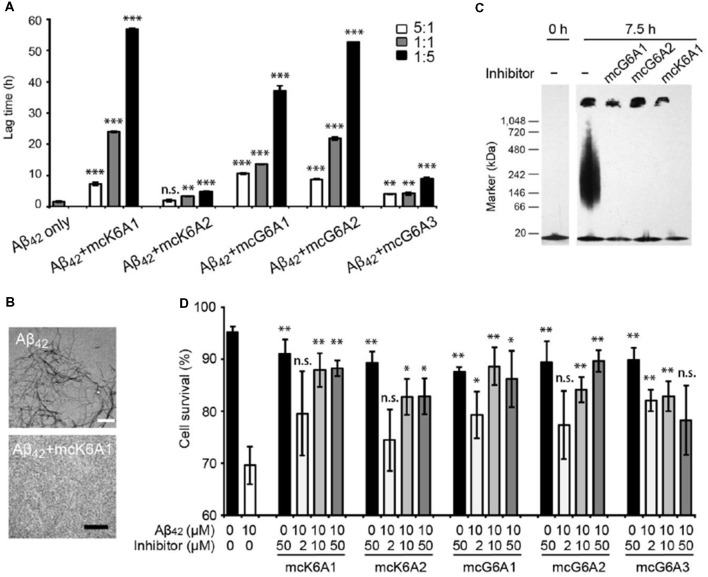
Inhibitory effects of designed macrocyclic peptides on Aβ_42_ amyloid aggregation and cytotoxicity. **(A)** The designed macrocyclic peptides, in particular mcK6A1, mcG6A1 and mcG6A2, significantly inhibit the amyloid fibril formation of Aβ_42_ in a dose-dependent manner. **(B)** Transmission electron microscopy (TEM) images of Aβ_42_ (20 μM) after incubation without inhibitors (top) and with 1.0 equivalent of mcK6A1 (bottom) to Aβ monome for 15 h. The scale bars are 200 nm. **(C)** Inhibition of Aβ_42_ oligomers. Aβ_42_ oligomers formed after 7.5 h of incubation at a concentration of 5 μM (by Aβ_42_ monomer equivalence) were invisible on the native gel with the addition of five molar excess of designed macrocyclic peptides. **(D)** The designed peptide inhibitors ameliorated Aβ_42_ cytotoxicity to PC-12 cells. The first column is the cells treated with 0.1 mM NaOH and phosphate buffer saline (PBS) as a positive control. Error bars correspond to standard deviations three replicates of each experiment. *p-value < 0.05; **p-value < 0.01; ***p-value < 0.001; n.s. represents “not significant”.

Next, we tested the inhibitory effects of the macrocyclic peptides on Aβ_42_ amyloid aggregation. The result showed that, in comparison with the free peptides, the macrocyclic peptides remarkably enhanced the inhibition on Aβ_42_ aggregation (Figures 2B, 4A,B, and Supplementary Figures S6–S10). For instance, the macrocycle carrying K6A1 (mcK6A1) is about 10 times more potent than free K6A1 in prolonging the lag time of Aβ aggregation. The macrocyclic peptides inhibited the amyloid aggregation of Aβ_42_ in a dose-dependent manner. McK6A1, mcG6A1 and mcG6A2 showed remarkably strong inhibition with a 7–10-fold increase of the lag time at sub-stoichiometric concentrations of 0.2 molar equivalence to Aβ_42_ monomer (Figure 4A).

Moreover, we found that the designed macrocyclic peptides can inhibit the formation of Aβ_42_ oligomers, the toxic intermediates of Aβ aggregation, monitored by the native gel (Figure 4C). This result demonstrated that targeting ^16^KLVFFA^21^ and ^37^GGVVIA^42^ can prevent both oligomer and fibril formation, indicating the potential important role of these two segments in the early stage of Aβ_42_ aggregation. To further assess whether the designed peptides can reduce Aβ cytotoxicity, we performed the MTT-based cell viability assay. The result showed that the designed macrocyclic peptides can significantly reduce the cytotoxicity of Aβ_42_ to PC-12 cells even with a molar ratio of inhibitor to Aβ_42_ as low as 0.2:1 (Figure 4D). Also, the designed macrocyclic peptides showed little toxicity to the PC-12 cells (Figure 4D). In addition, the designed inhibitors of Aβ_42_ showed no inhibition of the amyloid aggregation of other amyloid proteins (e.g., α-synuclein and the K19 variant of Tau), indicating that the designed peptides are highly sequence-specific (Supplementary Figure S11).

### Designed Peptides Selectively Inhibit the Aggregation of Aβ_42_ but Not Aβ_40_

**Figure 5:**
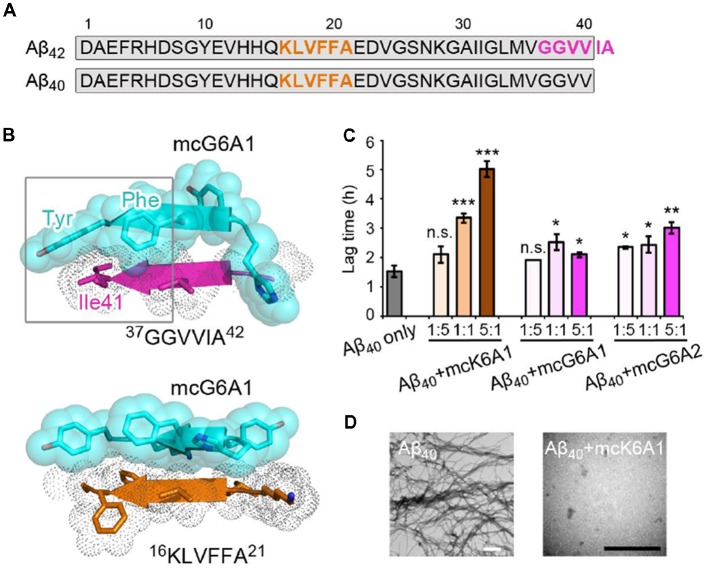
Specificity of designed macrocyclic peptides for the inhibition of Aβ_42_ and Aβ_40_ aggregation. **(A)** The sequences of Aβ_42_ and Aβ_40_. The amyloid-forming segment ^16^KLVFFA^21^ (highlighted in orange) is present in both Aβ_42_ and Aβ_40_, while segment ^37^GGVVIA^42^ (highlighted in magenta) is present only in Aβ_42_. The consensus sequence of Aβ_42_ and Aβ_40_ is highlighted in gray. **(B)** The structure models of mcG6A1 (cyan) in complex with ^37^GGVVIA^42^ (magenta) and ^16^KLVFFA^21^ (orange), respectively. McG6A1 was designed based on the structure of GGVVIA. Residues Tyr and Phe of mcG6A1, and Ile41 of GGVVIA (highlighted with a gray frame) engage in van der Waals interactions at the inhibitor-target interface. In contrast, mcG6A1 designed for GGVVIA has no specific side-chain interactions, but merely non-specific back-bone interactions with KLVFFA. **(C)** The effects of mcK6A, mcG6A1 and mcG6A2 on Aβ_40_ aggregation (30 μM by Aβ_40_ monomer equivalence), measured by ThT assay. Error bars correspond to standard deviations of three replicates of each experiment. *p-value < 0.05; **p-value < 0.01; ***p-value < 0.001; n.s. represents “not significant.” **(D)** TEM images of Aβ_40_ (30 μM) after incubation without inhibitors (left), and with 1.0 equivalent of mcK6A1 to Aβ monome (right). The scale bars are 200 nm.

Selective inhibition of Aβ_42_ aggregation over that of Aβ_40_ is challenging because Aβ_42_ is only two residues longer than Aβ_40_ at the C- terminus (Figure 5A). Since segment ^37^GGVVIA^42^ exists only in Aβ_42_, the designed peptides that target this segment may selectively inhibit the aggregation of Aβ_42_ but not that of Aβ_40_. As shown in the designed models, mcG6A1 that is designed to target ^37^GGVVIA^42^ forms extensive side-chain interactions with ^37^GGVVIA^42^ (Figure 5B). The aromatic residues Tyr and Phe of mcG6A1 interact with Ile41 of ^37^GGVVIA^42^ *via* van der Waals forces. The absence of Ile41 and Ala42 in Aβ_40_ diminishes the binding of mcG6A1 to Aβ_40_. Indeed, the experimental data showed that mcG6A1 and mcG6A2 that strongly inhibit the amyloid aggregation of Aβ_42_, cannot effectively inhibit the aggregation of Aβ_40_, as measured by ThT assay (Figure 5C, Supplementary Figures S12, S13). Note that a weak inhibitory effect of mcG6A1 and mcG6A2 to Aβ_40_ remains, which might come from non-specific backbone interactions between the inhibitors and Aβ_40_ (Figure 5B). In contrast, mcK6A1 that was designed to target the ^16^KLVFFA^21^ segment, a segment important for the amyloid aggregation of both Aβ_42_ and Aβ_40_, showed a dose-dependent inhibition of both Aβ_42_ and Aβ_40_ aggregation (Figures 4A, 5C,D, and Supplementary Figure S14). However, the inhibitory efficiency of mcK6A1 on Aβ_40_ is weaker than that on Aβ_42_, indicating that ^16^KLVFFA^21^ may play a more important role in Aβ_42_ aggregation than that of Aβ_40_. This implication is in agreement with the hypothesis that Aβ_42_ and Aβ_40_ may employ different amyloid nucleation and aggregation process (Sánchez et al., 2011; Meisl et al., 2014).

## Discussion

Development of peptide-based drugs is gaining greater attentions. In general, peptide-protein interactions have a high density of hydrogen bonds and highly complementary packing *via* hot-spot binding residues, leading to high binding affinity and exquisite selectivity with fewer off-target side effects (Kaspar and Reichert, 2013). Many attempts have been made to rationally design peptide inhibitors of amyloid protein aggregation, including modified internal segments of parent amyloid proteins, non-natural amino-acid inhibitors, proline substitutions, and other methods (Abedini et al., 2007; Sievers et al., 2011). Recently, RosettaDesign shows effectiveness for designing novel proteins and peptides with predicted structures having atomic accuracy (Bhardwaj et al., 2016; Huang et al., 2016). This technical advance has enabled the peptide inhibitor design of Tau aggregation (Abedini et al., 2007; Seidler et al., 2018). In this study, we designed peptides that can efficiently inhibit Aβ_42_ aggregation. Notably, the designed peptides show selectivity for the intended amyloid target, in contrast to small molecule inhibitors (e.g., EGCG and methylene blue) that broadly interfere amyloid aggregation of many proteins (Necula et al., 2007; Jiang et al., 2013; Palhano et al., 2013). Furthermore, the designed peptides can differentiate Aβ_42_ from Aβ_40_, demonstrating the accuracy and potency of structure-based rational design.

Short peptides composed of natural amino acids normally form unstructured ensembles in solution. If a defined conformation is required for target binding, conformational changes may occur upon binding, at a large entropic cost. This counteracts enthalpy gain from the favorable interaction of the designed peptide and its target, and consequently reduces the binding affinity of the peptide with its target. Therefore, constraining the designed peptide in the desired conformation (“pre-organization”) can minimize the entropic cost and increase the binding affinity. Chemical scaffolds provide a powerful toolbox for constraining peptides in defined secondary or tertiary structures in solution (Mowery et al., 2009; Azzarito et al., 2013; Cheng et al., 2013; Johnson and Gellman, 2013). In this work, we use a macrocyclic β-sheet mimic scaffold to constrain the designed peptides into β strands. Our results show significant enhancement of inhibition gained by the conformational constraint, which highlights the importance of conformation-constraint and the advantage of a chemical scaffold in the development of peptide binders. In addition, biopharmaceutical properties, such as degradation resistance and membrane permeability, may be achieved by modifying the chemical scaffold, rather than changing the inhibitor sequences.

Macrocyclic β-sheet mimics have been shown to be a useful model system to study the structural basis of amyloid-like oligomers and fibrils (Liu et al., 2012; Cheng et al., 2013; Zheng et al., 2013; Salveson et al., 2016). A variety of key amyloidogenic segments from different amyloid proteins (e.g., Aβ, α-synuclein and prion) were constructed into the macrocycles (Zheng et al., 2011; Cheng et al., 2012). However, the self-assembling and potential toxic properties of macrocyclic molecules that contain native amyloid-forming sequences hinder application of macrocycles in the development of amyloid inhibitors (Liu et al., 2012; Salveson et al., 2016). In this study, by using RosettaDesign approach, we developed novel sequences and incorporated them into macrocycles. These designed macrocyclic peptides resist self-assembly and exhibit little cytotoxicity. In additional to Aβ, the structures of many other pathogenic amyloid fibrils have been determined recently (Tuttle et al., 2006; Fitzpatrick et al., 2017; Murray et al., 2017). Thus, the strategy of combining RosettaDesign and chemical scaffolds may be useful for peptide inhibitor design of different amyloid proteins for a variety of amyloid-related diseases.

## Data Availability

All datasets generated for this study are included in the manuscript and/or the supplementary files.

## Author Contributions

All authors listed have made a substantial, direct and intellectual contribution to the work, and approved it for publication.

## Conflict of Interest Statement

The authors declare that the research was conducted in the absence of any commercial or financial relationships that could be construed as a potential conflict of interest.

## Footnotes

^1^https://www.rosettacommons.org
