# Supplementary material for: Structure-Based Peptide Inhibitor Design of Amyloid-β Aggregation
Source: Front Mol Neurosci. 2019 Mar 4;12:54. doi: 10.3389/fnmol.2019.00054 (PMC6409328; doi:10.3389/fnmol.2019.00054)
Supplement: Supplementary file 1 [file Data_Sheet_1.doc]

**Supplementary Information**

**Structure-based peptide inhibitor design of amyloid-β aggregation**

Jinxia Lu^1,¶^, Qin Cao^2, ¶^, Chuchu Wang^3,4,¶^, Jing Zheng^5^, Feng Luo^3,4^, Jingfei Xie^3,4^, Yichen Li^1^, Xiaojuan Ma^3,4^ , Lin He^1,5^, David Eisenberg^2^, James Nowick^6^, , Lin Jiang^7^,*, Dan Li^1^,*

^1^Key laboratory for the Genetics of Developmental and Neuropsychiatric Disorders (Ministry of Education), Bio-X Institutes, Shanghai Jiao Tong University, Shanghai 200030, China;

^2^UCLA-DOE Institute for Genomics and Proteomics, University of California, Los Angeles, CA 90095, USA;

^3^Interdisciplinary Research Center on Biology and Chemistry, Shanghai Institute of Organic Chemistry, Chinese Academy of Sciences, Shanghai 200032, China;

^4^University of Chinese Academy of Sciences, Beijing, China.

^5^Shanghai Center for Women and Children's Health, Shanghai 200062, China

^6^Department of Chemistry, University of California, Irvine, Irvine, California CA 92697-2025, USA.

^7^Department of Neurology, Easton Center for Alzheimer’s Disease Research, David Geffen School of Medicine, University of California, Los Angeles, California 90095, United States

^¶^ These authors contributed equally to this work.

*To whom correspondence should be addressed. E-mails: lidan2017@sjtu.edu.cn; jianglin@ucla.edu

**This file includes:**

**Supplementary Table 1**

**Supplementary Figures 1 to** **14**

**Reference: 1-5**

**Supplementary Table 1.** Secondary structure contents of designed peptide inhibitors in solution measured by CD spectroscopy. Data were analyzed by using CDPro^1^.

| Peptide ID | Sequence | Secondary structure (%) | | |
| --- | --- | --- | --- | --- |
|  |  | α-Helix | β-Sheet | Unstructured |
| K6A1 | TLWYK | 2.9 | 25.1 | 72.0 |
| K6A2 | EHWYH | 4.3 | 23.3 | 72.4 |
| G6A1 | HYFKY | 0.4 | 48.1 | 51.5 |
| G6A2 | HYYIK | 0.1 | 33.2 | 66.7 |
| G6A3 | KYYEI | 0 | 56.9 | 43.1 |


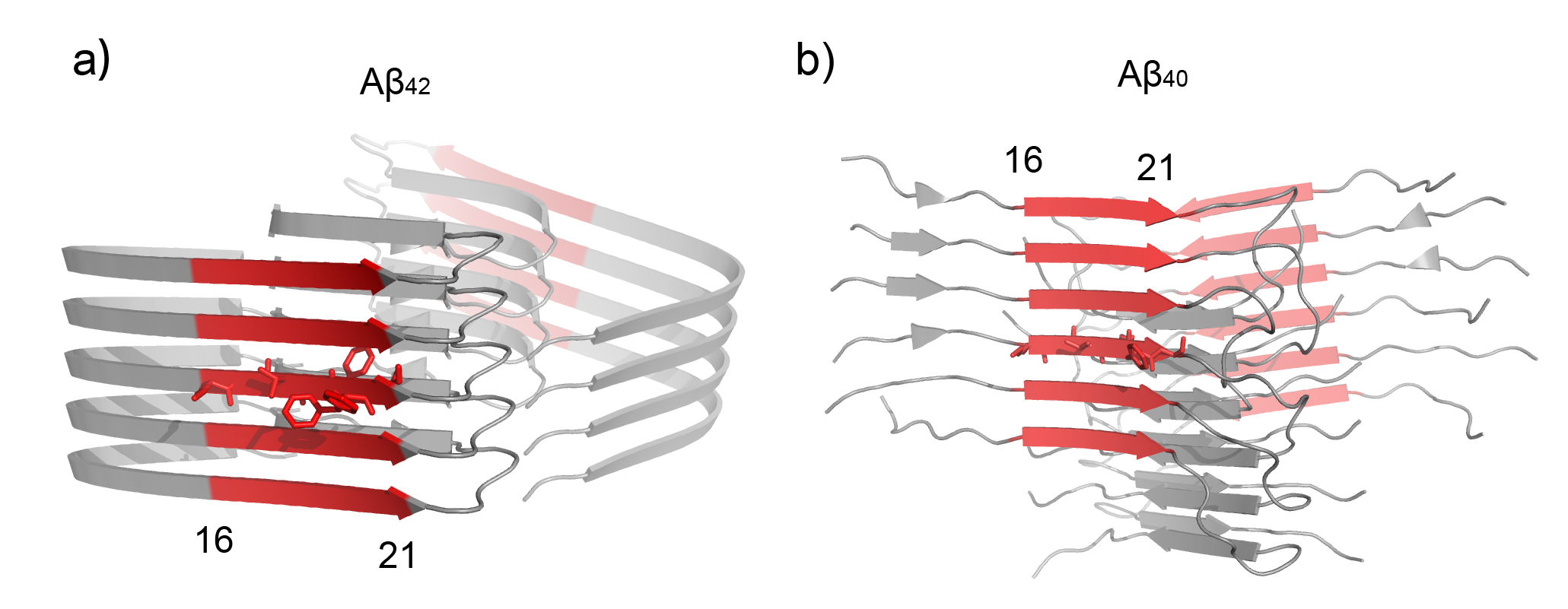


**Supplementary Figure 1.** Structures of segment ^16^KLVFFA^21^ in the full-length Aβ structures. (a) The structure of Aβ_42_ fibril determined by cryo-EM (PDB ID: 5OQV). (b) The structure of Aβ_40_ fibril determined by solid-state NMR (PDB ID: 2LMN). Segment ^16^KLVFFA^21^ is highlighted in red.


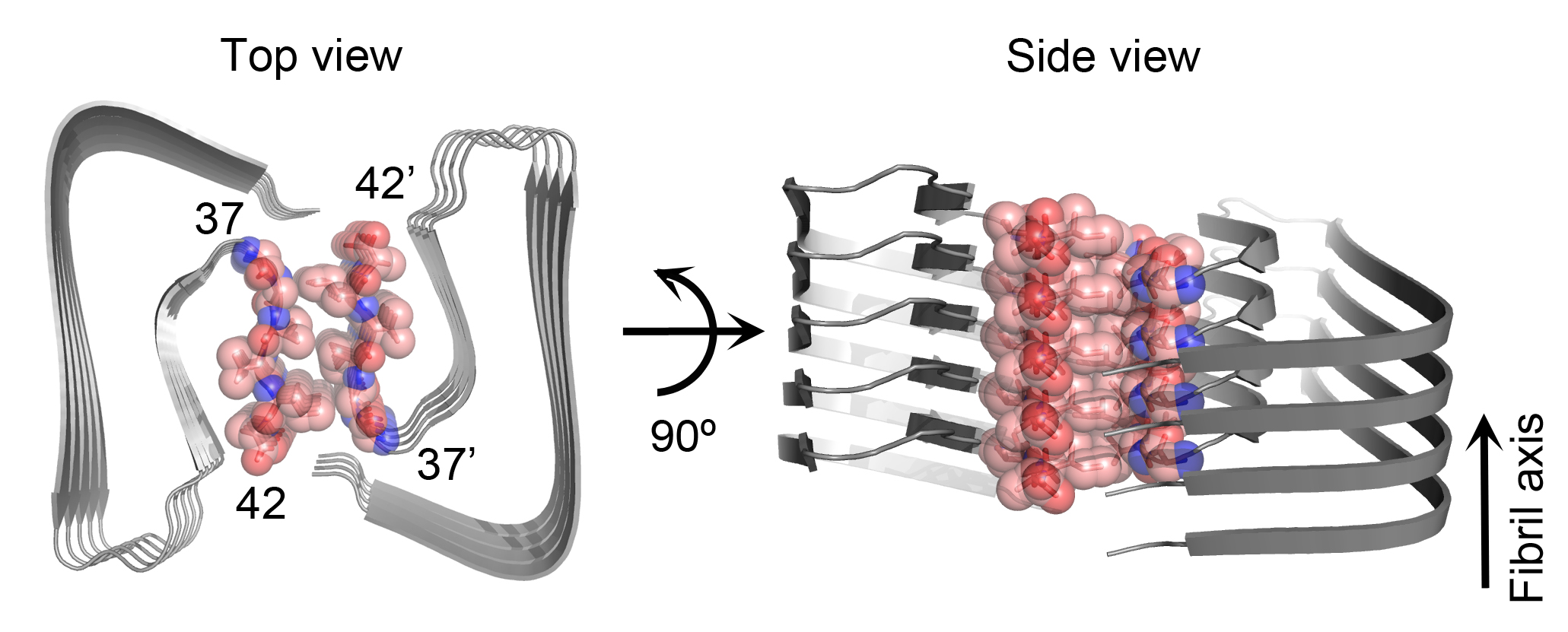


**Supplementary Figure 2.** The structure of segment ^37^GGVVIA^42^ in the fibril structure of full-length Aβ_42_ determined by cryo-EM (PDB ID: 5OQV). The ^37^GGVVIA^42^ segment is shown as sticks and spheres. Hydrophobic interactions between ^37^GGVVIA^42^ side chains contribute dominantly to the interface of the two protofilaments of the mature fibril.


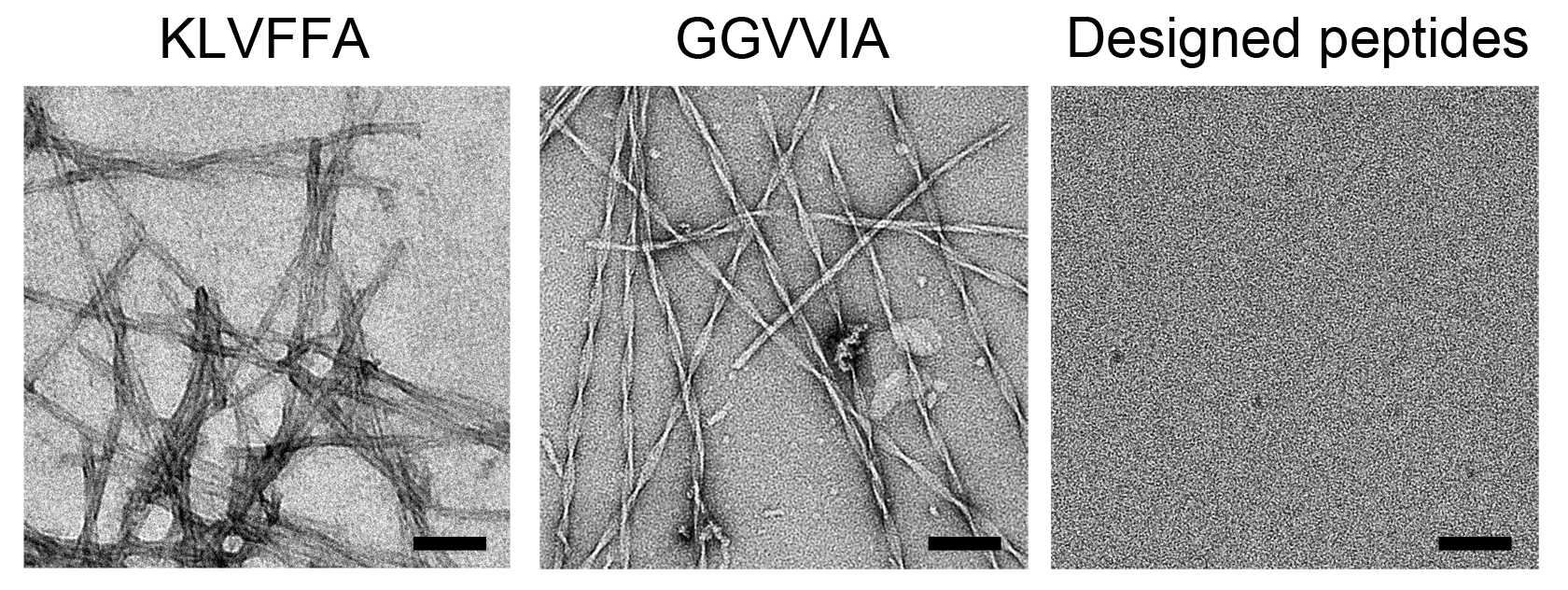


**Supplementary Figure 3.** TEM images of amyloid fibrils formed by target segments KLVFFA and GGVVIA. Fibrils were formed by dissolving 5 mg/ml of peptides with water and incubating at 37 ºC. The designed peptide inhibitors including free and macrocyclic peptides do not form fibrils. The scale bars are 100 nm.


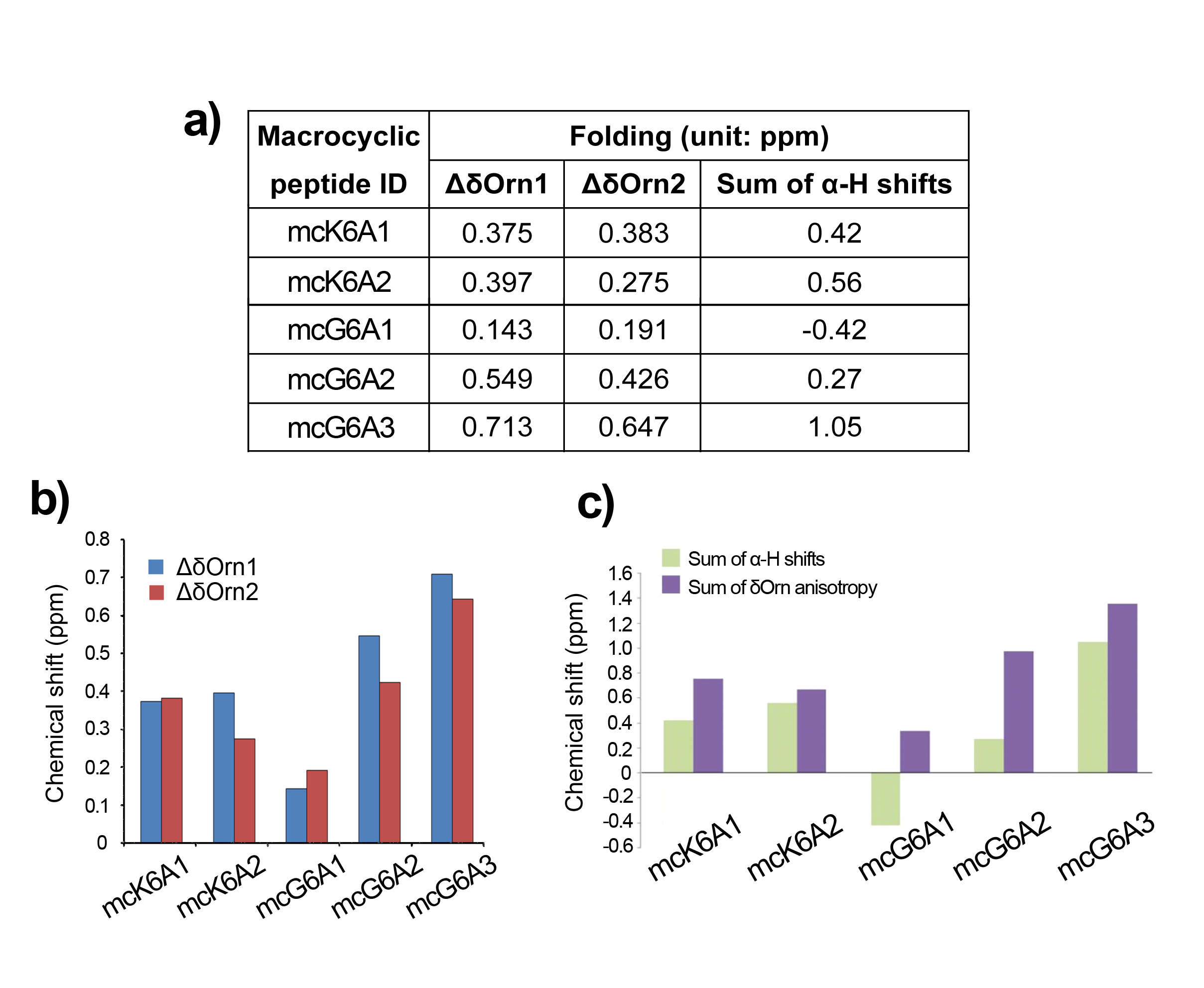


**Supplementary Figure 4.** ^1^H NMR study validates the designed macrocyclic peptide inhibitors generally adopt folded β-strand structures in solution. The degree of β-strand folding of macrocyclic peptides can be assessed by measuring the magnetic anisotropy of the diastereotopic δOrn δ-protons (ΔδOrn) and downfield shifting of the amino acid α-protons of the macrocycles^3-5^. (a) The values of α-H shifts and ΔδOrn anisotropy of the designed macrocyclic peptides indicates that mcG6A2, mcG6A3, mcK6A1 and mcK6A2 exhibit moderate to good folding, while mcG6A1 exhibit partial folding in solution. (b) The ΔδOrn1 and ΔδOrn2 anisotropy of each macrocyclic peptide. (c) The sum of α-H shifts and ΔδOrn anisotropy of each macrocyclic peptide. All NMR data were collected at the peptide concentration of 2 mM in D_2_O at 298 K.


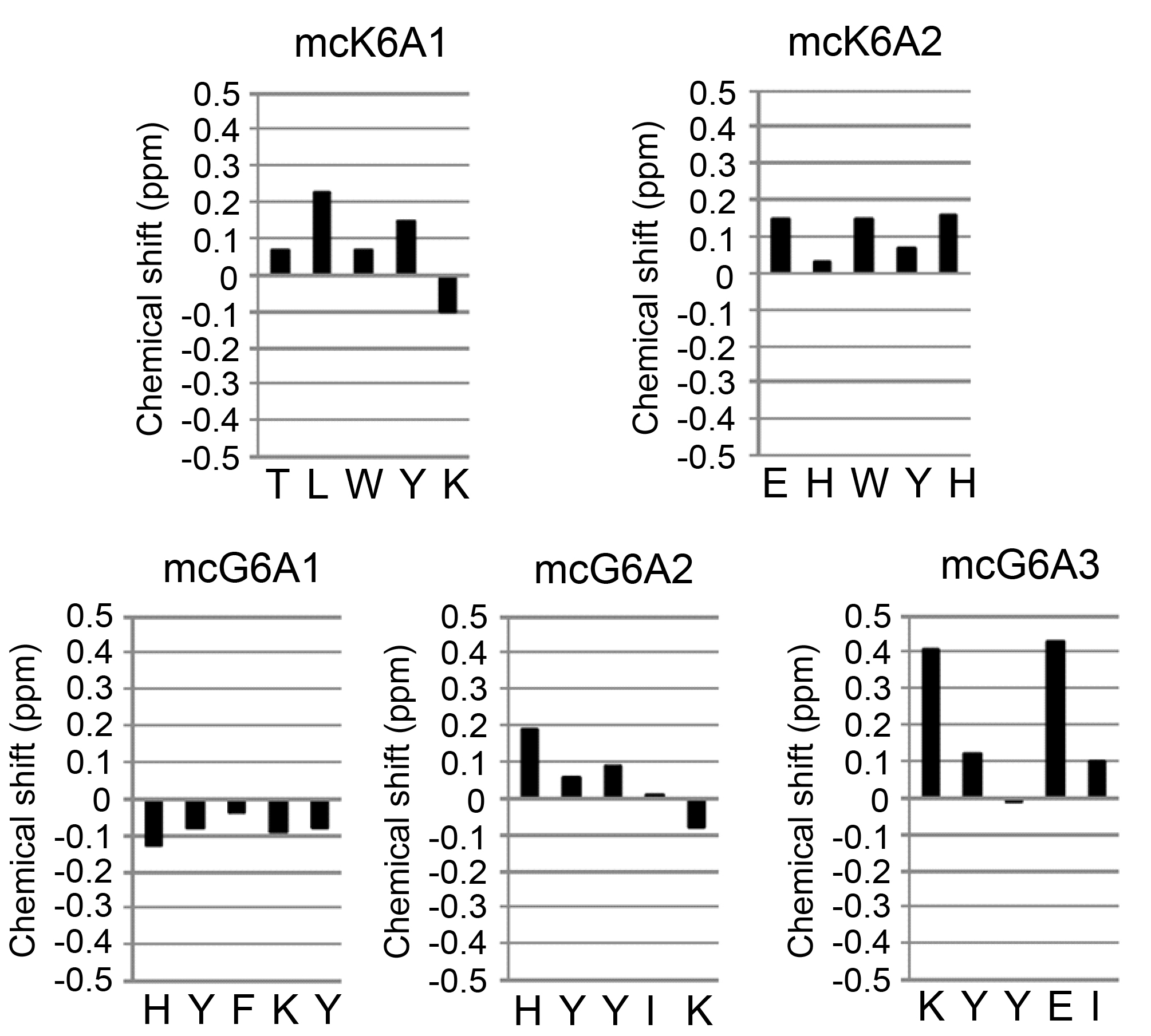


**Supplementary Figure 5.** Folding of macrocyclic peptides evaluated by the α-H shifts "fingerprint" of the individual amino acid of the inserted designed sequences. All NMR data were collected at the peptide concentration of 2 mM in D_2_O at 298 K.

**
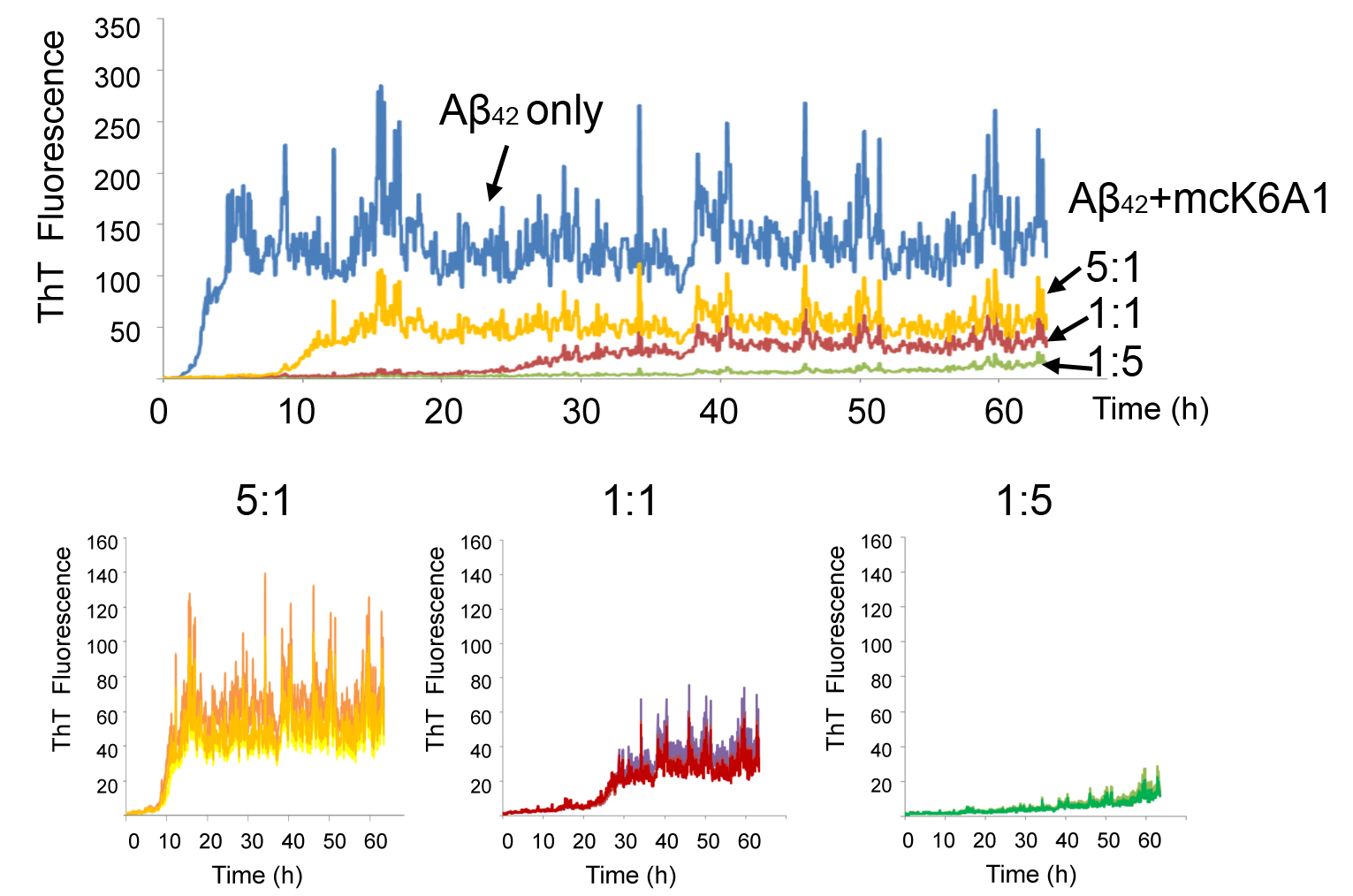
**

**Supplementary Figure 6.** Dose-dependent inhibition of mcK6A1 on Aβ_42_ aggregation measured by ThT fluorescence assay. Three replicates for each dosage are shown below at the bottom.

**
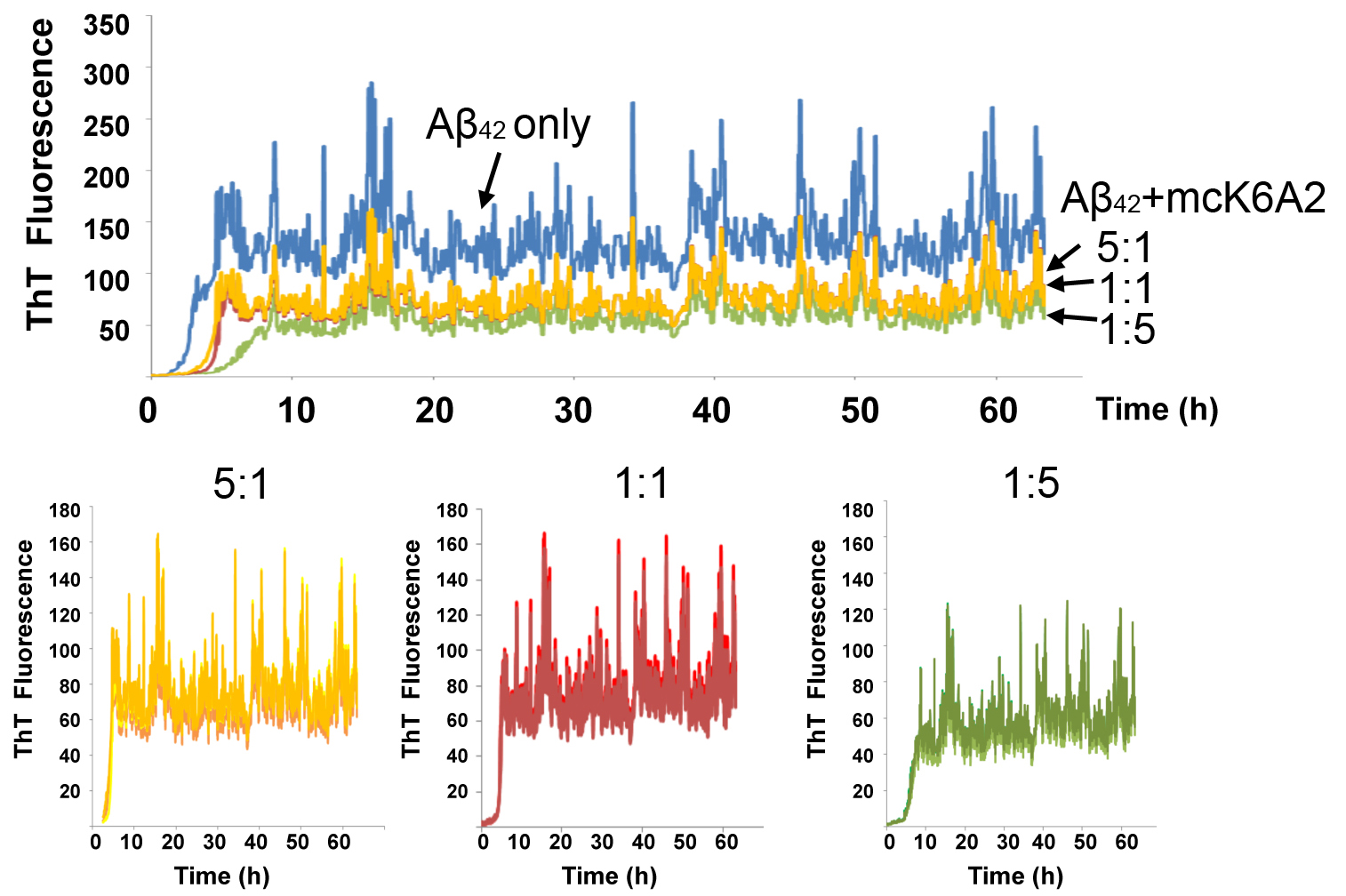
**

**Supplementary Figure 7.** Inhibition of mcK6A2 on Aβ_42_ aggregation measured by ThT fluorescence assay. Three replicates for each dosage are shown below at the bottom.


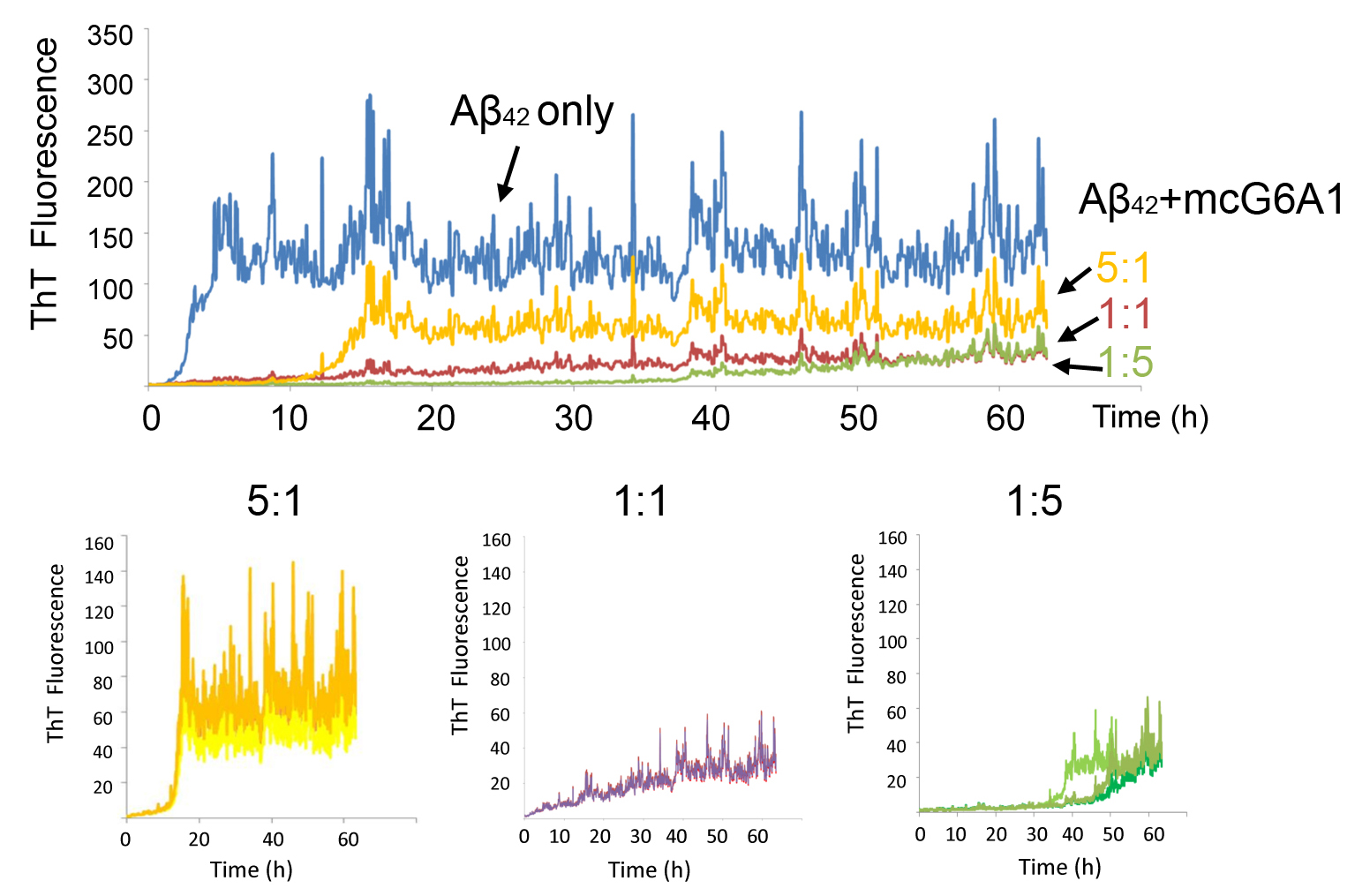


**Supplementary Figure 8.** Dose-dependent inhibition of mcG6A1 on Aβ_42_ aggregation measured by ThT fluorescence assay. Three replicates for each dosage are shown below at the bottom.


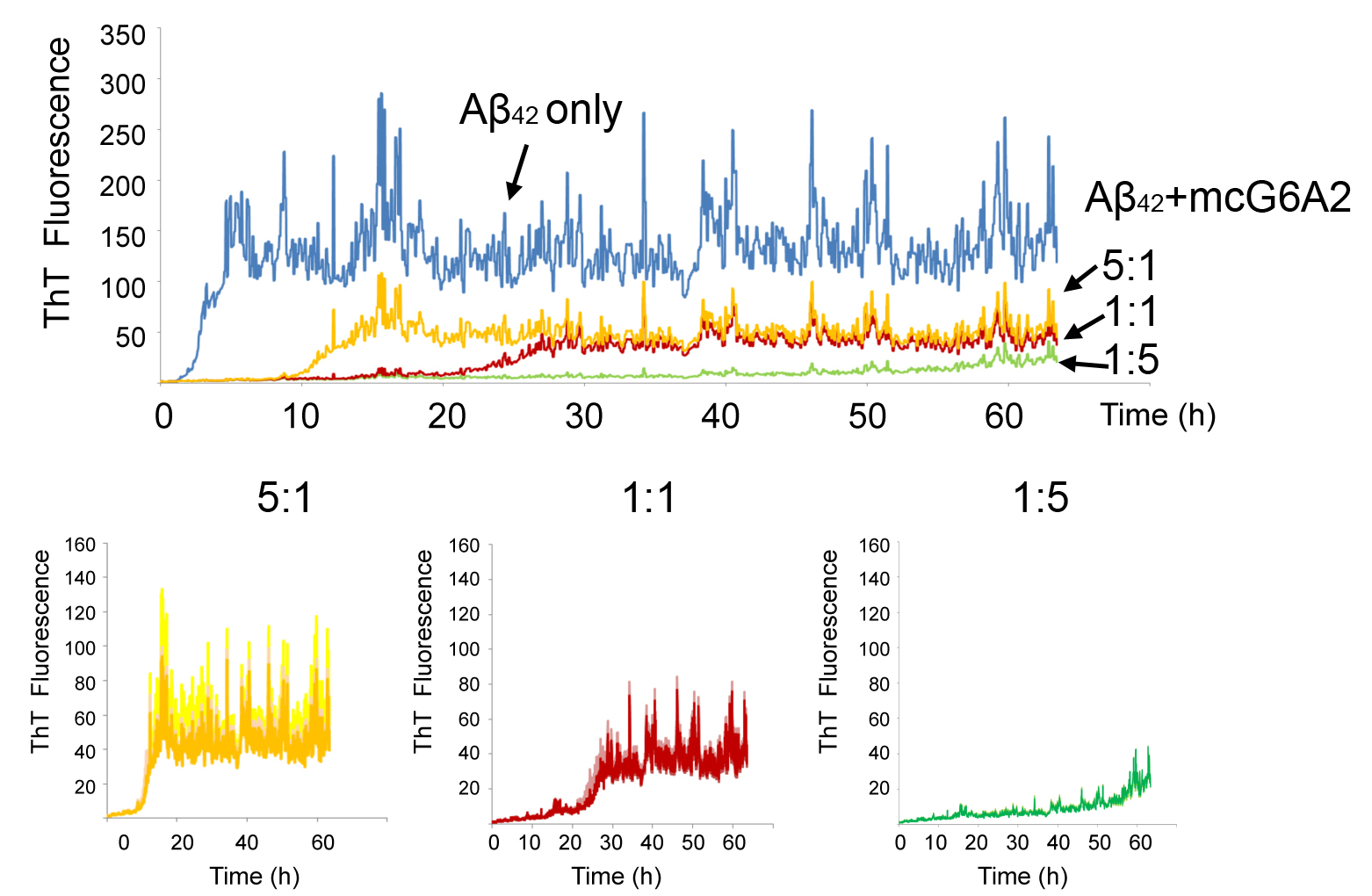


**Supplementary Figure 9.** Dose-dependent inhibition of mcG6A2 on Aβ_42_ aggregation measured by ThT fluorescence assay. Three replicates for each dosage are shown below at the bottom.

**
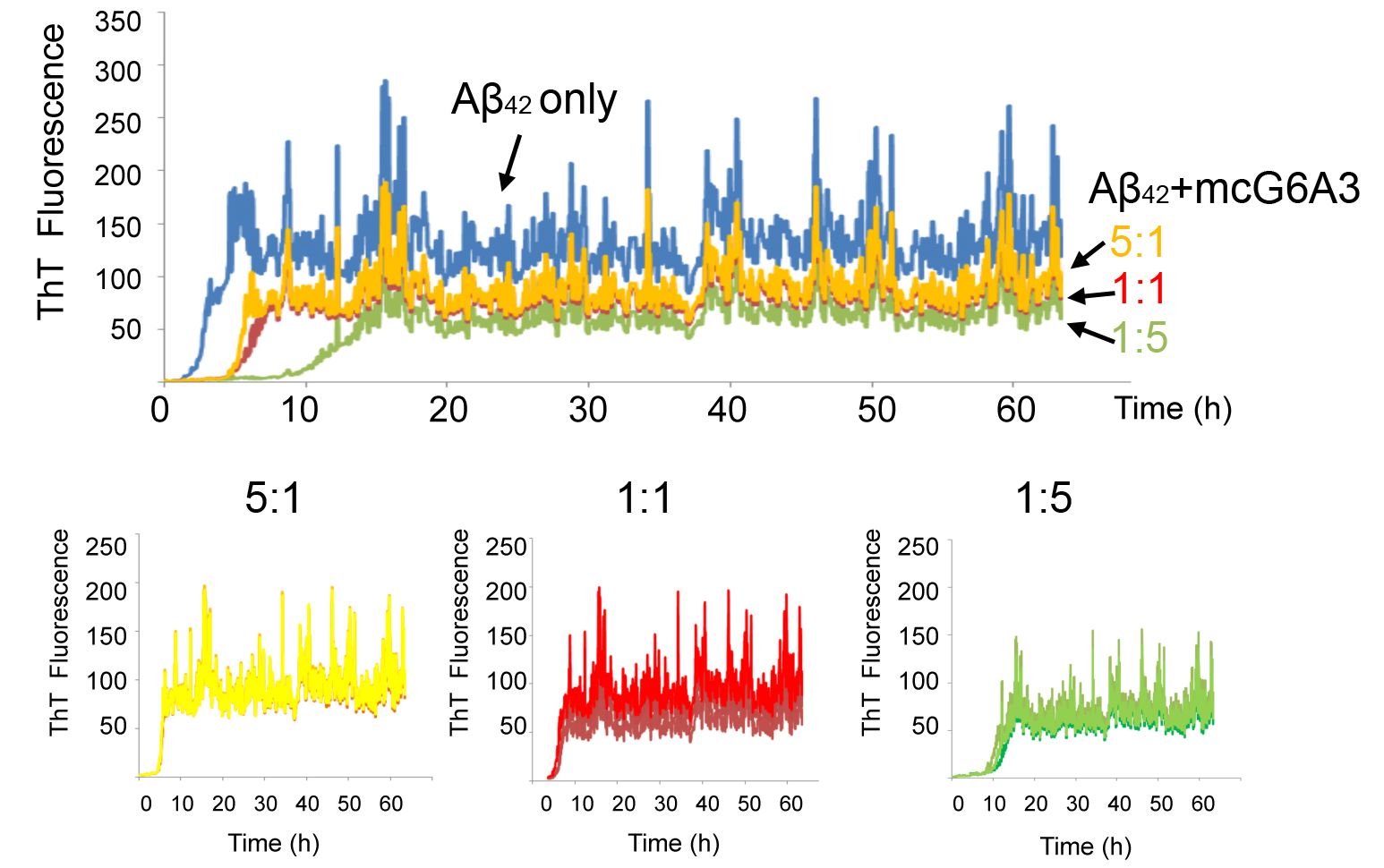
**

**Supplementary Figure 10.** Inhibition of mcG6A3 on Aβ_42_ aggregation measured by ThT fluorescence assay. Three replicates for each dosage are shown below at the bottom.

**
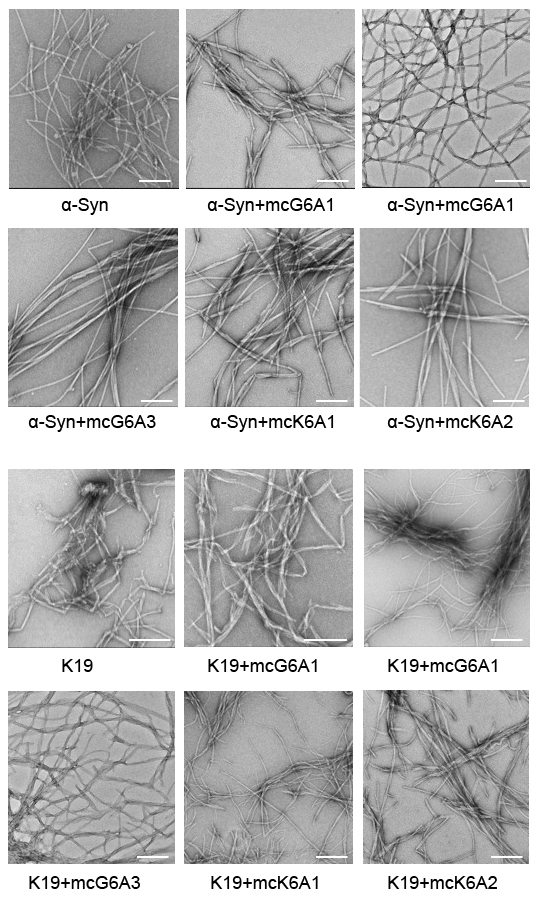
**

**Supplementary Figure 11.** Effects of designed peptides on the amyloid fibril formation of α-syn and tau K19. Peptides designed for inhibiting Aβ aggregation showed no significant influences on the amyloid fibril formation of α-syn and tau K19 imaged by TEM. α-Syn and tau K19 (50 μM) were incubated with 1.0 equiv. of designed peptides for 15 h. The scale bars are 200 nm.


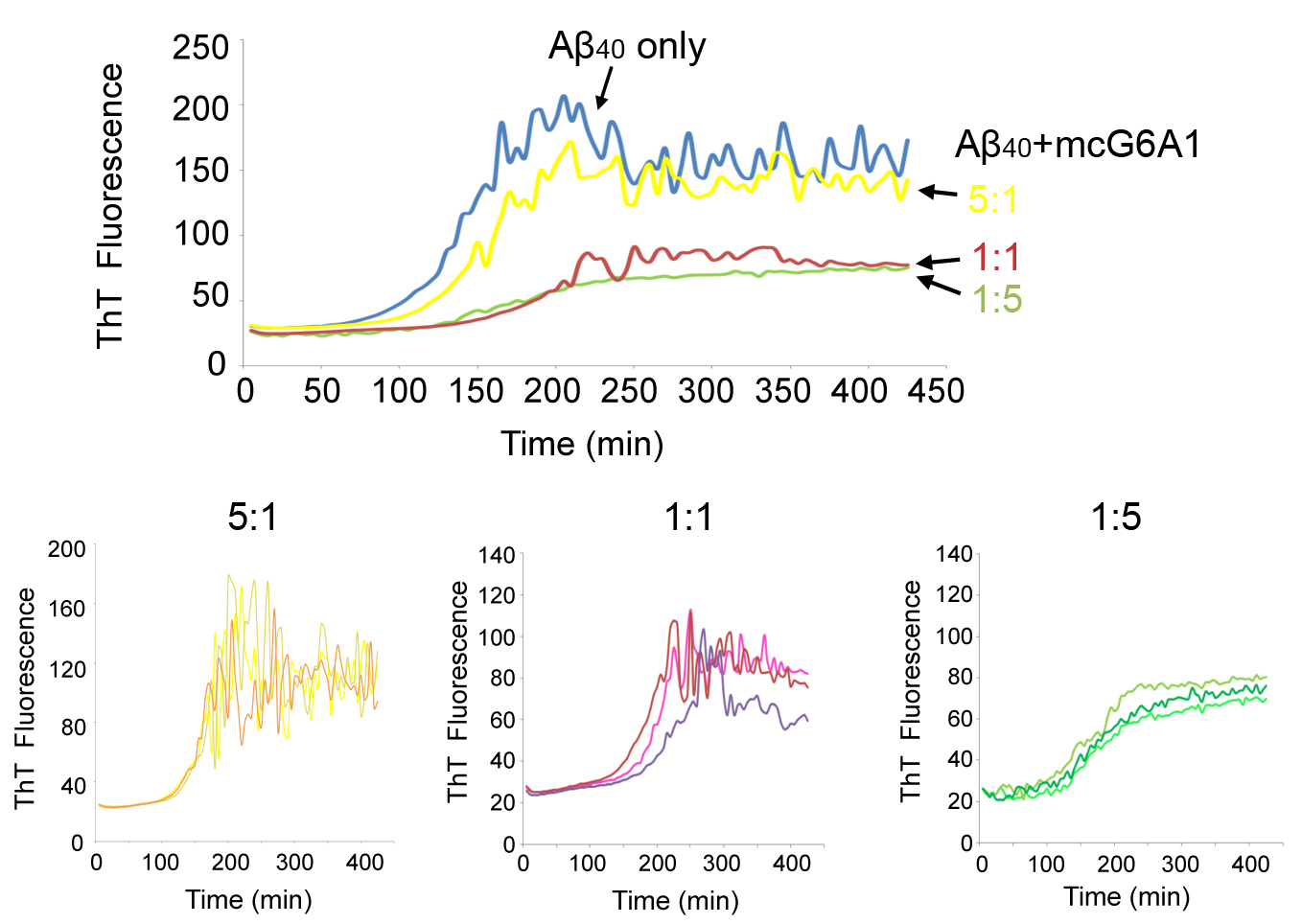


**Supplementary Figure 12.** Effect of mcG6A1 on Aβ_40_ aggregation measured by ThT fluorescence assay. Three replicates for each dosage are shown below at the bottom.

**
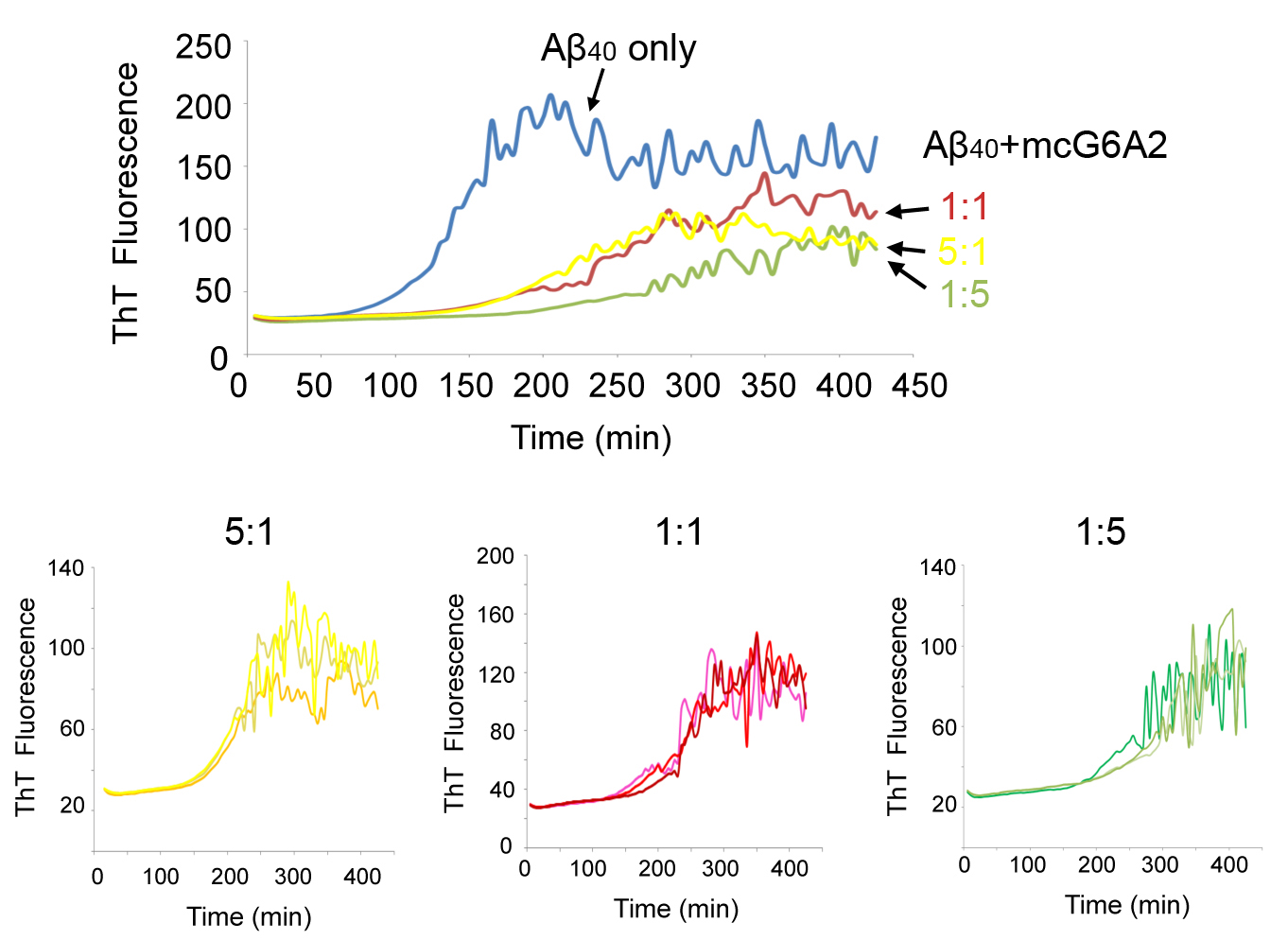
**

**Supplementary Figure 13.** Effect of mcG6A2 on Aβ_40_ aggregation measured by ThT fluorescence assay. Three replicates for each dosage are shown below at the bottom.

**
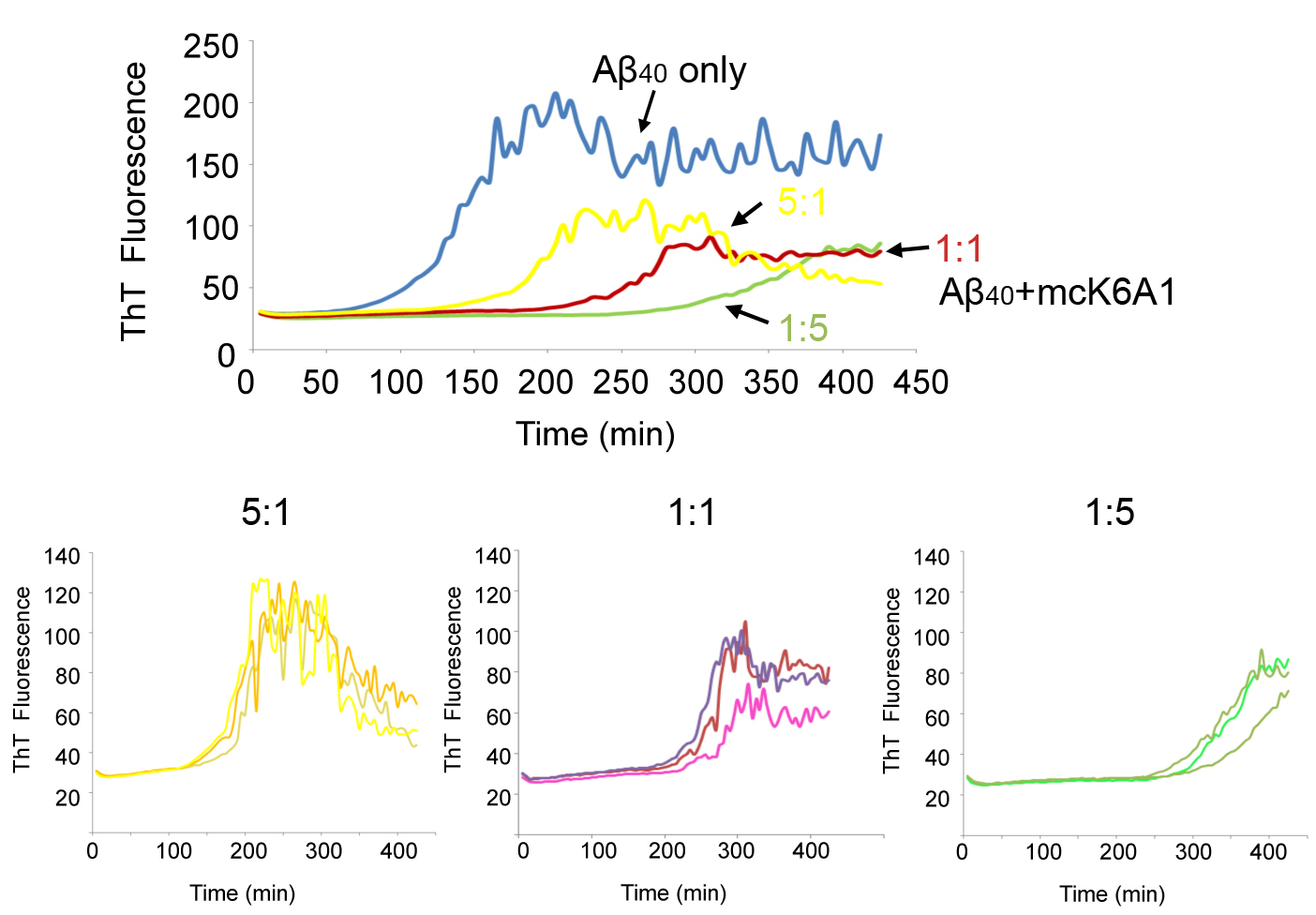
**

**Supplementary Figure 14.** Dose-dependent inhibition of mcK6A1 on Aβ_40_ aggregation measured by ThT fluorescence assay. Three replicates for each dosage are shown below at the bottom.

**References**

[1] W. C. Johnson, *Proteins* **1999**, *35*, 307-312.

[2] V. H. Finder, I. Vodopivec, R. M. Nitsch, R. Glockshuber, *J. Mol. Biol.* **2010,** *396,* 9-18.

[3] J. Zheng, C. Liu, M. R. Sawaya, B. Vadla, S. Khan, R. J. Woods, D. Eisenberg, W. J. Goux, J. S. Nowick, *J. Am. Chem. Soc.* **2011**, *133*, 3144-3157.

[4] T. V. Khasanova, O. Khakshoor, J. S. Nowick, *Org. Lett.* **2008**, *10*, 5293-5296.

[5] S. Levin, J. S. Nowick, *Org. Lett.* **2009**, *11*, 1003-1006.
